# Harnessing digital twins for public health surveillance and pandemic preparedness

**DOI:** 10.1186/s12919-026-00397-x

**Published:** 2026-09-21

**Authors:** Hossein Hassani, Barbara Tornimbene, Francisco J. Doblas-Reyes, Julia Fitzner, Steve MacFeely

**Affiliations:** 1 International Institute for Applied Systems Analysis (IIASA), Vienna, Austria; 2 World Health Organization Hub for Pandemic and Epidemic Intelligence, Berlin, Germany; 3 Barcelona Supercomputing Center (BSC), Barcelona, Spain; 4 Data and Analytics Division, World Health Organization, Geneva, Switzerland

**Keywords:** Digital twins, Health care, Innovation, Data governance, Privacy, Ethics

## Abstract

The 10th session of the WHO Pandemic & Epidemic Intelligence Innovation Forum focused on the transformative potential of digital twins in enhancing public health systems and pandemic preparedness. Digital twins, virtual replicas of physical entities or systems, can integrate real-time data to simulate and predict outcomes, providing valuable insights for public health interventions. This session explored their applications in healthcare, urban planning, and climate adaptation, while addressing challenges such as data governance, ethical considerations, and system interoperability. Discussions also emphasised the need for overcoming limitations and leveraging multisectoral collaboration to maximise the utility of digital twins in advancing global health resilience.

## Introduction

With advancements in data science, artificial intelligence (AI), and the Internet of Things (IoT) [1], which is a network of interconnected physical devices that collect, exchange, and process data through the internet, digital twins have emerged as sophisticated tools capable of mirroring physical systems, processes, and environments. In the context of public health, they have the potential to enhance disease surveillance, optimise healthcare delivery, and improve emergency response planning.

The WHO Hub for Pandemic and Epidemic Intelligence (WHO Hub in Berlin) convened its 10th Innovation Forum on June 20, 2024, to discuss digital twins and their applications in pandemic and epidemic intelligence. A leading scientist from the International Institute for Applied Systems Analysis (IIASA), Vienna, Austria, contributors from the Barcelona Supercomputing Center (BSC), the WHO Data and Analytics division, and from the Collaboration for Advance Analytics (CAA) Unit at the WHO Hub in Berlin, shared insights into the evolving landscape of digital twin technology and its relevance for pandemic preparedness and public health surveillance.

The session’s objectives were threefold: to define the concept and explore the applications of digital twins, to identify their limitations, and to discuss ethical and data governance considerations in their use for public health. By examining real-world examples and current research, the forum highlighted the transformative potential of this technology to enhance decision-making and resource allocation in health emergencies.

### The concept and evolution of digital twins

IIASA introduced digital twins as dynamic, virtual replicas that integrate real-time data to provide a comprehensive digital representation of physical objects, processes, or systems [2]. The concept traces its origins to NASA’s efforts in the 1960 s, where "mirroring technology" was developed to simulate spacecraft behaviour and minimise risks during missions [3, 4]. This foundation evolved into digital twins, officially coined in 2000, which have since expanded beyond aerospace to industries such as manufacturing, healthcare, and urban planning, peaking during the COVID-19 pandemic [5–7].

Digital twins differ fundamentally from static models or simple 3D representations by incorporating continuous feedback loops between the physical and digital realms. They mirror the real-time status, condition, or position of the physical counterpart. There are different types of digital twins, including Component Twins, Asset Twins, Process Twins, and System & Network Twins, each designed for specific applications [8]. Data serves as their backbone, enabling real-time modelling and decision-making by continuously integrating information from various sources. Sensors embedded in mobile devices, hospital equipment, environmental monitoring systems, and urban infrastructure collect and transmit data on variables such as temperature, movement, patient vitals, and air quality, ensuring that digital twins remain accurate and dynamically responsive to changing conditions.

IIASA emphasised that digital twins are not just AI models. While traditional models rely on fixed inputs and produce defined outputs, digital twins are continuously evolving systems that integrate real-time data, generative AI, machine learning, predictive analytics, and cloud computing to create accurate virtual replicas of physical objects, processes, or systems. They require continuous human oversight, expert validation, and real-world feedback loops. The latter allows for continuous refinement and improvement of the virtual representation. The information and the advanced analytics enable digital twins to learn and reason so that they can simulate scenarios, identify patterns, and optimise performance. This shift represents a broader movement from abstract simulation to integrated, interactive system replication—an important conceptual foundation for their use.

### The use of digital twins across different sectors

Digital twins are transforming multiple sectors by enabling real-time simulation, predictive analytics, and data-driven decision-making. Their ability to integrate diverse data sources, model complex systems, and provide continuous feedback loops makes them invaluable for improving infrastructure, environmental resilience, and industrial operations.

The Barcelona Supercomputing Center (BSC) demonstrated how digital twins are playing a critical role in climate adaptation, offering a new level of precision and responsiveness in predicting and mitigating climate-related risks. By simulating complex interactions between climate variables and human systems, digital twins provide detailed projections on temperature trends, precipitation shifts, and extreme weather events. The Climate Adaptation Digital Twin at BSC leverages advanced computational models to assess climate risks across multiple sectors [9]. This initiative goes beyond standard climate projections by incorporating AI-driven climate pattern recognition, and infrastructure modelling. Its use of real-time data streaming and high-performance computing enables simulations to be continuously updated with the latest information about anthropogenic emissions and satellite observations. This feature enhances the accuracy and responsiveness of adaptation planning, ensuring that policies remain adaptive as climate conditions evolve. These advanced models facilitate the integration of high-resolution climate simulations with environmental, infrastructural, and socioeconomic data, allowing decision-makers to move from reactive crisis management to proactive adaptation strategies where sectors such as energy, water management, and urban planning can optimise their responses to droughts, water shortages, and ecosystem changes.

In urban planning, these models provide decision-makers with dynamic forecasting capabilities that help strengthen infrastructure resilience [10]. IIASA stressed how, by aggregating data from sensors embedded in roads, buildings, and public transit systems, digital twins provide real-time insights that improve urban mobility, reduce congestion, and support efficient energy consumption. Cities can test how different adaptation strategies impact long-term sustainability, such as redesigning transportation networks to cope with heat stress, planning new green spaces to improve air quality, or modifying building materials to withstand extreme temperature fluctuations. The ability to assess future risks and adaptation outcomes in real time makes digital twins a transformative tool for climate resilience, helping societies better navigate the growing complexities of a changing climate. For instance, BSC’s climate adaptation digital twin can analyse rising temperatures and urban heat islands, allowing cities to speed up the assessment of how heatwaves affect different neighbourhoods, identify at-risk populations, and optimise heat mitigation strategies such as urban greening and cooling infrastructure. Similarly, digital twins can simulate flood risks by integrating rainfall projections with topographical and hydrological data, helping urban planners design better stormwater drainage systems, flood barriers, and emergency response plans.

Beyond urban applications, digital twins are reshaping industrial operations, particularly in manufacturing, aerospace, and energy sectors [7]. IIASA highlighted how industries leverage digital twins for predictive maintenance, process optimisation, and efficiency gains. In aerospace engineering, for example, digital twins facilitate continuous monitoring of aircraft components, allowing for early detection of potential failures and reduced downtime. Similarly, in energy production, they optimise power grid performance, renewable energy integration, and demand forecasting, ensuring more reliable and sustainable energy distribution. These applications not only reduce operational costs but also support environmental sustainability goals by minimising waste and maximising efficiency.

The WHO Data and Analytics division further explored how digital twins improve supply chain resilience and crisis management by modelling potential disruptions, optimising logistics, and streamlining resource allocation [11]. Digital twins provide real-time visibility into supply chain operations, enabling organisations to anticipate bottlenecks, assess geopolitical risks, and develop contingency plans. In disaster response, digital twins can simulate wildfires, floods, or infrastructure breakdowns, allowing emergency teams to allocate resources efficiently and coordinate large-scale interventions. Their predictive capabilities ensure that supply chains remain agile and responsive to unexpected events, reinforcing global resilience in critical sectors.

### Applications in public health and pandemic preparedness

Digital twins can also have transformative potential in public health, particularly in pandemic preparedness, disease surveillance, and healthcare optimisation [12]. These sophisticated virtual models can provide public health officials with an advanced tool to anticipate crises, manage resources more efficiently, and implement evidence-based interventions.

The WHO Data and Analytics Division showed how, at the individual level, digital twins can be used to integrate patient data from electronic health records, wearable sensors, and genomic profiling, providing a dynamic, continuously updating health model that allows for personalised treatment plans and early disease detection [13, 14]. This personalised approach has significant implications for precision medicine, as clinicians can test different treatment options on a virtual replica before applying them in real life, minimising risks and improving outcomes.

Beyond individualised care, digital twins can extend to hospital and healthcare system management, where they can simulate patient flow, optimise bed occupancy, and predict resource needs, ensuring facilities remain agile and prepared during health emergencies [12, 15]. In a pandemic scenario, such models could anticipate surges in hospital admissions, track ICU capacity, and identify supply chain vulnerabilities, allowing administrators to adjust operations in real time. One of the greatest advantages of digital twins is their ability to move healthcare systems from reactive crisis management to proactive planning, reducing inefficiencies and ensuring preparedness for large-scale outbreaks.

Expanding the concept beyond clinical care, digital twins could also hold immense potential in epidemiological modelling and public health surveillance. They can continuously assimilate real-time inputs from diverse sources, such as mobility patterns, environmental factors, and clinical case reports, enabling more accurate forecasting of outbreaks. However, as flagged by the WHO CAA Unit, their use in large-scale public health interventions remains in its early stages. This is partly due to the multifactorial nature of public health, which requires the integration of social behaviour, climate data, economic factors, and epidemiological trends into a unified model. Unlike isolated digital twins in industry, public health applications must account for diverse data streams, making implementation more challenging. By simulating the interactions between populations, healthcare infrastructure, and policy interventions, though, digital twins could help authorities evaluate the effectiveness of containment measures before they are implemented, ensuring that public health responses are both evidence-based and adaptive to changing conditions. To achieve this, the discussions during the meeting reinforced the idea that public health digital twins should integrate multiple domains.

## Towards integrated systems: a collaborative path forward

Despite the many challenges associated with digital twin implementation, the forum consistently emphasised multisectoral collaboration as a vital pathway forward. Given the complexity, their successful deployment in public health depends on interdisciplinary coordination. This includes active participation from public health agencies, academic researchers, technology firms, urban planners, and policymakers, all working together to develop shared governance frameworks, interoperability standards, and technical protocols.

Cross-sector collaboration ensures that digital twins are not only technically robust but also context-sensitive, inclusive, and adaptable to the realities of diverse communities. This is particularly important for pandemic intelligence, where health outcomes are influenced by a convergence of factors such as urban density, pollution levels, climate stressors, and mobility patterns. Integrating digital twins developed for cities or climate adaptation with those for health systems can offer a more holistic view of public health risks and guide timely, informed interventions.

During the panel discussion, IIASA and the WHO CAA Unit emphasised that large-scale implementation must be approached incrementally. One proposed strategy is to develop “sentinel” digital twins—targeted models focused on specific regions, systems, or diseases—as proof-of-concept pilots [16]. These smaller-scale initiatives can be rigorously tested and refined before scaling up to more complex, integrated ecosystems. This approach provides a manageable entry point for institutions seeking to experiment with digital twins while building technical and governance capacity over time.

Examples from Singapore, Dubai, and Helsinki, where digital twins are already being used to manage traffic, optimise energy use, and enhance emergency preparedness, offer valuable lessons. These urban-scale models illustrate how the integration of infrastructure and environmental data into a digital ecosystem can also be leveraged to monitor air quality, assess heat stress, and simulate disease transmission, ultimately contributing to resilient and responsive public health systems.

## Addressing challenges and limitations

Despite their promise, the implementation of digital twins in public health faces significant challenges related to data security, interoperability, computational requirements, ethical governance, and technical capacity [17–19]. Throughout the forum, experts underscored the need to address these systemic barriers to enable the meaningful application of digital twins in pandemic preparedness and response.

A key concern was data security, privacy, and user trust. As digital twins increasingly integrate sensitive and diverse data, from clinical records to environmental sensors and behavioural indicators, the risks of unauthorised access, misuse, and breaches become more acute. Public confidence in these systems will depend on their transparency, validation mechanisms, and governance structures, which must clearly define how data is used, protected, and shared.

Compounding this is the fragmented nature of public health data, which is often siloed across institutions and formatted inconsistently. The WHO CAA Unit highlighted how the lack of interoperability constrains digital twins’ ability to ingest real-time data and respond effectively to outbreaks. Enabling data integration and interoperable platforms is essential for linking digital twins across sectors such as smart cities, climate adaptation, and epidemiological surveillance.

Forum participants also expressed concern about the opacity of AI-enhanced digital twins, which—like many advanced AI systems—can become “black boxes” without deliberate design choices to ensure auditability, traceable data lineage, and stakeholder accountability. Clear model assumptions, robust evaluation frameworks, and channels for expert and community feedback are vital to preserve trust and interpretability, especially when digital twins inform high-stakes public health decisions.

The use of generative AI was recognised as a promising way to improve digital twin simulations and scenario modelling. However, this also introduces new risks: AI-driven bias may emerge if training datasets are skewed toward high-income contexts, leading to models that perform poorly for low-resource settings. Mitigating this requires careful data curation and sustained investment in ethical AI governance.

In addition to governance and interoperability, infrastructure and access disparities pose significant challenges. BSC noted that high-resolution digital twins, such as those used for climate adaptation or pandemic simulation, demand computational resources and infrastructure that many public health institutions do not possess. Without dedicated support, this technological gap risks widening health inequities, particularly if under-represented regions are excluded from model development or decision frameworks.

Another pressing issue is the shortage of specialised expertise. Building and managing digital twins requires knowledge in AI, data science, epidemiology, and computational modelling, skills that are often in short supply in public health agencies. BSC highlighted that many qualified professionals migrate to the private sector due to more competitive salaries and career opportunities, leaving public institutions at a disadvantage. Addressing this skills gap will require investment in training, retention, and interdisciplinary collaboration.

Ensuring that digital twin systems are accurate, equitable, and aligned with public health goals will depend on ongoing dialogue, inclusive policy frameworks, and global cooperation. The forum reinforced the central role of multilateral organisations in convening expertise, setting standards, and supporting knowledge exchange. With sustained collaboration across disciplines such as climate science, public health, and digital engineering, digital twins can mature into an integrated infrastructure for real-time public health intelligence and long-term preparedness.

## Conclusion

The 10th session of the WHO Pandemic & Epidemic Intelligence Innovation Forum underscored the transformative potential of digital twins to enhance public health surveillance, pandemic preparedness, and climate adaptation. By integrating real-time data and fostering interdisciplinary collaboration, digital twins offer a pathway toward more agile, informed, and responsive decision-making processes. Their capacity to simulate complex, evolving systems opens new possibilities for improving resource allocation, infrastructure resilience, and the design of public health interventions. The forum also recognised that achieving these benefits will depend not only on technological innovation but on structured pathways for implementation, such as beginning with sentinel models and scaling through phased integration.

A central insight from the dialogue was the need to move beyond siloed, single-purpose digital twin applications toward more integrated and adaptive ecosystems. Rather than attempting to build comprehensive models from the outset, a more pragmatic approach would begin with focused, sentinel digital twins targeting specific diseases, regions, or systems and progressively expanding in complexity and scope. This incremental path allows for testing, validation, and refinement, building confidence and trust in the technology while setting the stage for broader applications.

In parallel, the discussants in the forum emphasised the importance of enabling data integration to allow seamless exchange and interpretation across different systems. Collaboration among experts in public health, data science, urban planning, climate research, and technology development is essential to ensure digital twins are developed and deployed in ways that are context-sensitive, operationally feasible, and socially responsible. Investments in scalable computing infrastructure, capacity building, and cross-sector coordination will be vital to support the growth and integration of digital twin technologies.

The future of digital twins in public health also calls for a broader shift in practice. Public health systems have traditionally relied on periodic data collection and aggregate analyses. The capabilities introduced by digital twins, particularly their use of continuous, high-resolution data streams, will offer the opportunity to move toward more timely, granular, and adaptive intelligence. This shift could enable more effective targeting of interventions, especially for vulnerable or underserved populations, and enhance the ability to respond to emerging threats with speed and precision.

As technological, institutional, and methodological capacities mature, digital twins are poised to become a core component of global health intelligence. Yet their success will depend not only on innovation but also on the thoughtful alignment of technical feasibility, ethical integrity, equitable infrastructure investment, and inclusive governance. Developing digital twins that benefit all populations fairly demands transparent data-sharing practices, legal safeguards, and continuous validation to avoid unintended bias or misuse, especially when handling sensitive health information.

With sustained commitment, digital twins could be an important component of public health security and preparedness, ensuring that decision-makers have access to rapid scenario modelling that leads to improved decision-making, transforming global health preparedness into a more adaptive, precise, and equitable practice.

## Data Availability

Not applicable.

## Abbreviations

AI: Artificial intelligence
BSC: Barcelona Supercomputing Center
CAA: Collaboration for Advanced Analytics
ICU: Intensive care unit
IIASA: International Institute for Applied Systems Analysis
IoT: Internet of Things
NASA: National Aeronautics and Space Administration
WHO: World Health Organization
